# Neural plasticity impairment in chronic post-surgical hypoparathyroidism: a cross-sectional and prospective pilot study

**DOI:** 10.1530/EC-26-0020

**Published:** 2026-03-26

**Authors:** Andrea Palermo, Gaia Tabacco, Anda Mihaela Naciu, Eleonora Sargentini, Monica Cereghino, Gabriella Musumeci, Maria Valeria Di Lazzaro, Mariagrazia Rossi, Davide Norata, Antonio Todisco, Licia Maria Celani, Alessandro Cruciani, Giulia Massa Rolandino, Francesco Motolese, Fioravante Capone

**Affiliations:** ^1^ Unit of Metabolic Bone and Thyroid Diseases, Fondazione Policlinico Universitario Campus Bio-Medico, Rome, Italy; ^2^ Unit of Endocrinology and Diabetes, Campus Bio-Medico University, Rome, Italy; ^3^ Department for the Promotion of Human Science and Quality of Life, San Raffaele Open University, Rome, Italy; ^4^ Unit of Neurology, Fondazione Policlinico Universitario Campus Bio-Medico, Rome, Italy

**Keywords:** hypoparathyroidism, hypocalcemia, PTH, neural plasticity

## Abstract

**Background:**

Despite adequate conventional therapy, patients with chronic post-surgical hypoparathyroidism (HypoPT) frequently report persistent physical, emotional, and cognitive symptoms that affect quality of life, despite no overt cognitive impairment. The neurophysiological mechanisms underlying these complaints remain unclear.

**Purpose:**

To assess cortical excitability and plasticity in HypoPT patients using transcranial magnetic stimulation (TMS) and to explore the effects of parathyroid hormone (PTH) therapy on these parameters.

**Methods:**

We conducted a cross-sectional study including 32 HypoPT patients on stable conventional treatment without significant cognitive impairment and 16 age-matched healthy controls. In a prospective observational phase, a subgroup of six HypoPT patients (3-palopegteriparatide and 3-teriparatide) was reassessed after 48 months of PTH therapy. All participants underwent TMS evaluation, including motor evoked potentials (MEPs), short-latency afferent inhibition (SAI), short-interval intracortical inhibition (SICI), and assessment of plasticity using intermittent theta burst stimulation (iTBS). The primary outcome was the change in MEP amplitude after iTBS as an index of cortical plasticity.

**Results:**

MEP changes following iTBS differed significantly between HypoPT patients and controls (−0.02 vs 0.4 mV; *P* = 0.003; corrected *P* = 0.042), indicating reduced cortical plasticity in HypoPT. In contrast, HypoPT patients receiving long-term PTH therapy showed a significant increase in MEP amplitude, comparable to healthy controls. No other TMS measures differed significantly.

**Conclusion:**

This pilot study provides preliminary evidence of impaired cortical plasticity in HypoPT in the absence of overt cognitive impairment. Although limited by small sample size, the findings suggest that PTH therapy may modulate cortical plasticity. Further research is needed to clarify the clinical relevance and potential central nervous system effects of PTH in HypoPT.

## Introduction

Chronic hypoparathyroidism (HypoPT) is a medical condition characterized by low calcium levels in the blood, accompanied by low or inappropriately normal levels of parathyroid hormone (PTH). The conventional treatment involves the use of activated vitamin D and/or calcium supplements, but it does not fully mimic the proper action of PTH (1, 2, 3, 4). Indeed, despite adequate and balanced calcium and vitamin D supplementation, many HypoPT patients continue to experience symptoms causing a decline in their quality of life (QoL) (5, 6, 7, 8, 9). In particular, it has been shown that HypoPT is associated with disabling symptoms such as fatigue, cognitive disturbances (e.g. ‘brain fog’, difficulty in concentrating, and memory impairment), and emotional struggles (e.g. depression and anxiety) (5, 10). However, the pathogenesis of these symptoms, their impact on QoL impairment, and their connection to biochemical management or other disease aspects are not yet well understood.

PTH and PTH-related peptide (PTHrP) receptors have been identified in the central nervous systems (CNS), namely in neurons, reactive astrocytes, and endothelial cells (11), although their precise function remains unclear. Recently, Sikjaer *et al.* have found that the reduced QoL and cognitive performance, particularly in processing speed, executive functions, and memory, was associated with a smaller hippocampal volume in HypoPT patients (12). These data suggest that HypoPT can be associated with measurable cognitive impairment and structural brain changes that correlate with the symptoms reported by patients (13); however, the underlying mechanisms remain unclear. Notably, PTH replacement therapy (3, 14, 15) – particularly with palopegteriparatide (16, 17, 18) – has been associated with improvements in overall QoL among patients with HypoPT.

Neurophysiological tools can be used to investigate, non-invasively, the pathogenetic mechanisms in different forms of cognitive impairment (19). In particular, transcranial magnetic stimulation (TMS) is a powerful tool to study, *in vivo*, brain circuits, as it allows one to assess several CNS properties, such as excitability, plasticity, and connectivity. Recently, TMS has been applied to patients with dementia, enabling the identification of potential markers of the pathophysiology and predictors of cognitive decline (20). Single-pulse protocols allow testing the excitability of cortical neurons, while paired-pulse protocols (a ‘conditioning’ stimulus precedes a suprathreshold ‘test’ stimulus with a variable interstimulus interval) allow testing the functionality of specific excitatory or inhibitory circuits. Finally, TMS delivered in repetitive modality (rTMS) is used to test the plasticity of cortical synapses since it can induce prolonged changes of cortical excitability (20). Using these TMS protocols, it is possible to obtain several neurophysiological parameters related to brain activity, both in physiological and in pathological conditions. Recent evidence suggests that, in different cognitive disorders, TMS parameters can be altered even in the preclinical stage of the disease, before clinical symptoms emerge (20), thus suggesting that the impairment of cortical plasticity and excitability, measured by TMS, could represent a specific pathogenetic mechanism of cognitive dysfunction rather than a generic epiphenomenon of neurodegeneration. Our hypothesis is that, similarly in HypoPT patients, cortical circuits explored through TMS are early altered, even in the absence of cognitive deficits.

Accordingly, the aim of the present study is to investigate, non-invasively, the functionality of brain circuits and networks in HypoPT patients without cognitive impairment. In particular, using TMS, we have compared cortical excitability and plasticity parameters in HypoPT patients and healthy controls, and we have evaluated the effect of PTH therapy on these features.

## Materials and methods

### Study population

The entire study population was enrolled from the outpatient clinic of Unit of Metabolic Bone and Thyroid Disorders, Fondazione Policlinico Universitario Campus Bio-Medico from January 2021 to December 2024. We enrolled 32 consecutive patients with chronic post-surgical HypoPT. The diagnosis of chronic post-surgical HypoPT was established at least 12 months post-surgery on the basis of clinical and biochemical features (low or inappropriately low PTH levels with low calcium levels on at least two prior occasions separated by an interval of at least 30 days). All patients must have been on stable regimens of supplemental calcium carbonate and vitamin D for at least 3 months prior to enrollment.

Subjects were excluded if they met any of the following criteria: i) they suffered from diabetes mellitus, hyperthyroidism, uncontrolled hypothyroidism, severe chronic liver or renal disease (glomerular filtration rate <30 mL/min), Cushing’s syndrome, sarcoidosis, rheumatic diseases (e.g. systemic lupus erythematosus or rheumatoid arthritis), or neurological disease, were heavy smokers, or had evidence of bone metastases; ii) they took drugs that could interfere with calcium metabolism or potentially affect nervous system function; iii) they took drugs that could interfere with the functioning of the nervous system; iv) they took or had taken PTH (1–34) or PTH (1–84) before the enrollment; v) they had serum magnesium levels below the lower limits or above the upper limits of normal on at least two prior occasions; and vi) they do not have a clinical significant cognitive deficit.

Moreover, 16 healthy, age- and sex-matched control subjects without any impairment of calcium, phosphate, and PTH concentration were consecutively recruited based on the exclusion criteria, including drug history, from the outpatient clinic of endocrinology, where they were referred for unrelated diseases (thyroid nodules with euthyroidism).

### Study design

All participants who fulfilled the inclusion/exclusion criteria and signed informed consent were recruited. In the cross-sectional evaluation, all subjects underwent the following procedures: i) clinical and biochemical assessment, ii) cognitive evaluation, and iii) neurophysiological study.

Moreover, a subgroup of HypoPT patients (*n* = 6, 18.7%) have been involved in a prospective evaluation aimed to test the impact of PTH treatment on neuroplasticity. This subgroup of HypoPT patients were neurophysiologically study 48 months after initiation of PTH treatment (3/6 subjects have been treated with palopegteriparatide and 3/6 with teriparatide). Indications to initiate PTH treatment were gastrointestinal side effects related to conventional therapy.

### Clinical and biochemical evaluation

We assessed the clinical characteristics of the entire study population by recording the medical history and reviewing the clinical, laboratory, and imaging results already performed on patients. Physical examination was performed in all subjects.

All biochemical measurements have been performed at the central laboratory of the Fondazione Policlinico Universitario Campus Bio-Medico. Fasting blood sampling was obtained in the morning (from 8:00 to 9:00 AM). Serum total calcium (normal: 8.4–10.2 mg/dL) and albumin were measured using automated methods (colorimetric assay). Serum calcium was adjusted for albumin (21). Serum thyroid-stimulating hormone, serum phosphate, and creatinine were also measured by automated techniques. The estimated glomerular filtration rate (eGFR) was calculated using the CKD-EPI equation. Intact PTH was measured by an immunochemiluminometric assay using the automatic analyzer Modular E170 (Roche Diagnostics, USA). Normal serum iPTH levels ranged between 13 and 85 pg/mL. The intra-assay coefficient of variation ranged from 2.2 to 3.4%, and the inter-assay CV ranged from 3.1 to 6.5%.

### Cognitive evaluation

In order to rule out a clinically significant cognitive impairment, the cognitive status of participants was evaluated through phone interviews. In particular, we employed a validated screening tool designed for remote cognitive assessment, the telephone-based Mini-Mental State Examination version 1 (ITEL-MMSE1) (22). The ITEL-MMSE is a 22-point questionnaire based on the standard MMSE, a neuropsychological tool assessing several functions, but it is designed for telephone administration. It includes different domains, including temporal (5 points) and spatial orientation (4 points), encoding (3 points), attention (5 points), recall (3 points), object naming (1 point), and sentence repetition (1 point). Studies comparing ITEL-MMSE with in-person MMSE demonstrate moderate to high correlation, supporting its validity as a screening and monitoring tool for cognitive impairment (23). Although a universally validated cutoff for the ITEL-MMSE has not been established, previous studies have suggested that scores below 18/22 correspond to MMSE scores of ≤24/30, a threshold commonly used to indicate cognitive impairment (22).

### Neurophysiological evaluation

Both patients and healthy controls underwent a standardized neurophysiological assessment of cortical excitability and plasticity, using TMS protocols including motor threshold determination, motor evoked potentials (MEPs), and paired-pulse paradigms (SAI, SICI, and ICF). All TMS measures were acquired following the recommendations of international expert guidelines (24) to ensure methodological rigor and reproducibility. We compared TMS-related parameters between patients and healthy controls, and subsequently in a subgroup of patients, we analyzed changes in TMS parameters before and 48 months after the initiation of PTH therapy.

Magnetic stimulation was performed using a high-power Magstim 200 magnetic stimulator (Magstim Co, UK), connected with a figure-of-eight coil positioned over the dominant motor cortex at the optimal scalp position to elicit motor responses in the contralateral first dorsal interosseous (FDI) muscle. The coil was positioned tangentially over the scalp at a 45° angle relative to the central sulcus, ensuring that the induced current flowed in a posterior-to-anterior direction through the cortex. Surface muscle responses were recorded using two 9-mm-diameter Ag/AgCl electrodes, with the active electrode placed over the motor point of the FDI muscle and the reference electrode positioned on the metacarpophalangeal joint of the index finger. The muscle responses were amplified and filtered (bandwidth: 3 Hz–3 kHz) using D360 amplifiers (Digitimer, UK). Data were acquired on a computer at a sampling rate of 10 kHz per channel and stored for subsequent offline analysis using a CED 1401 A/D converter (Cambridge Electronic Design, UK).

Prior to each experiment, the optimal site for eliciting MEPs in the right or left FDI muscles (i.e. the hotspot) was identified, after which the resting and active motor thresholds (RMT and AMT, respectively) of the dominant hemisphere were determined.

For each participant, different TMS protocols were used to test different neurophysiological parameters: i) short-interval intracortical inhibition (SICI), ii) intracortical facilitation (ICF), iii) short-latency afferent inhibition (SAI), and iv) theta burst stimulation (TBS).

SICI, which serves as a measure of GABAergic activity (25), involves delivering two magnetic stimuli through the same coil to evaluate the impact of the conditioning stimulus on the test stimulus. In this study, the conditioning stimulus was set at an intensity 5% below the AMT, while the test stimulus was adjusted to evoke a 1-mV MEP in the target muscle, with an interstimulus interval (ISI) of 2 and 3 ms. ICF, an index of glutamatergic activity (25), was assessed using an ISI of 15 ms. Ten stimuli were delivered in a randomized order for each ISI and as test stimuli (without a conditioning stimulus). Both SICI and ICF were expressed as the ratio between the amplitude of the conditioned MEP and the amplitude of the test MEP.

SAI is a measure of cholinergic activity obtained by pairing electrical stimulation of a peripheral nerve (conditioning stimulus) with TMS of the motor cortex (test stimulus) (26). Conditioning stimulus to the median nerve was given at the wrist through an electrical stimulator (Digitimer model DS7A, Digitimer, UK) using a bipolar electrode (cathode proximal) and an intensity inducing a painless thumb twitch. The intensity of the conditioning stimulus was set just above the motor threshold required to evoke a visible twitch in the thenar muscles, while TMS was adjusted to elicit a 1-mV MEP in the target muscle. The interstimulus intervals (ISIs) between the conditioning stimulus and TMS were individually determined based on the cortical response (N20) latency of somatosensory evoked potentials (SEPs), recorded from the dominant hand for each participant. SEPs were recorded with the active electrode attached on the scalp over P3 and reference on the contralateral P3, according to the international 10–20 system. Five hundred responses were averaged to identify the latency of the N20 peak. Peripheral electrical stimulation at the wrist for evaluating SEPs was delivered with the same setting described above for electrical stimulation of SAI. Three ISIs were investigated: i) N20 + 2 ms, ii) N20 + 3 ms, and iii) N20 + 4 ms. Ten stimuli were delivered in a randomized order at each ISI, as well as in a condition where TMS was delivered without a preceding conditioning stimulus. For SAI, the ratio of the grand average of the conditioned MEP (across all ISIs) to the test MEP was calculated. This was done because we have previously demonstrated that even if SAI is evident at all the tested ISIs, the grand mean has the advantage of reducing variability (27). The presentation order of the different conditions was randomized across subjects in both experiments, while MEPs were monitored online throughout the experimental session.

TBS is a rTMS protocol consisting of bursts of three TMS stimuli delivered at 50 Hz, repeated every 200 milliseconds, corresponding to the theta frequency (5 Hz). Intermittent TBS (iTBS) is a specific excitatory protocol in which 2-s trains of bursts are repeated every 10 s for a total duration of 190 s. As established in the literature, iTBS enhances corticospinal excitability, with its effects persisting beyond the stimulation period for at least 15 min. Multiple lines of evidence suggest that iTBS induces long-term potentiation (LTP)-like effects in the targeted neuronal populations. This is supported by the fact that TBS mimics the naturally occurring firing patterns of hippocampal neurons, which oscillate at 5 Hz (28). To assess plasticity propensity in our cohort, we recorded 15 MEPs at 120% of the RMT before (MEP_pre) and immediately after (MEP_post) the iTBS protocol. In addition, to quantify the magnitude of change induced by iTBS, we calculated the difference between MEP_post and MEP_pre, defining this variable as MEP_diff.

### Ethics approval and consent to participate

Our protocol adhered to the Declaration of Helsinki and the International Conference on Harmonization Good Clinical Practice, receiving approval from Campus Bio-Medico University Ethics Committees (Prot.:78/20PAR ComEt CBM). All participants provided informed consent, allowing the use of their anonymized information for data analysis. The corresponding author had full access to all the data in the study and took responsibility for its integrity and data analysis.

### Statistical analysis and sample size calculation

Sample size was estimated to ensure adequate power for both between-group and within-subject comparisons. As no prior data were available on TMS parameters specifically in patients with chronic post-surgical hypoparathyroidism, for the cross-sectional comparison between HypoPT subjects and healthy controls, we assumed a large effect size (Cohen’s *d* ≈ 1.2). Under these assumptions, a minimum of 10 participants per group would be required to achieve 80% power at a two-tailed significance level of 0.05. For the longitudinal evaluation of PTH therapy in HypoPT patients, the sample size calculation was based on published data on the efficacy of palopegteriparatide in improving QoL (18). Assuming a comparable standardized effect size (∼1.5) for neurophysiological outcomes reflecting central nervous system involvement, a minimum of six patients would be sufficient to detect within-subject changes with 80% power (*α* = 0.05). While this calculation was based on data from palopegteriparatide, we included both teriparatide- and palopegteriparatide-treated patients in the analysis based on their shared ability to activate PTH receptors, including PTH2R, which may be relevant for CNS effects.

Sample characteristics were described with median and interquartile range (IQR) or mean and standard deviation (SD) for quantitative variables and with absolute and relative (percentages) frequencies for qualitative ones. Data were analyzed for normality of distribution using the Shapiro–Wilk test. Between-group comparisons (HypoPT vs controls) for the parameters of interest – i.e., SICI, SAI, ICF, and MEP_diff – were performed using the Wilcoxon rank-sum test (Mann–Whitney *U*). Within-group comparisons (before vs after PTH therapy) were performed using the Wilcoxon signed-rank test. Effect sizes for the primary outcome (MEP_diff) were calculated using the rank-biserial correlation.

Bonferroni correction was applied to adjust for multiple comparisons for the cross-sectional evaluation. Due to the very small sample size (*n* = 6) and the exploratory intent of the prospective evaluation part of the study, no formal correction was applied. Statistical significance was set at *P*-values of less than 0.05, and data analysis was carried out through STATA (version 17, StataCorp, USA).

## Results

### Demographic and biochemical features

Demographic and biochemical data of HypoPT patients are reported in Table 1. The study population consisted of a total of 48 adults (38 females and 10 males). We included 32 HypoPT patients (26 females and 6 males with a mean age of 50 ± 12.7 years) and 16 healthy control subjects (12 females and 4 males with a mean age of 45.4 ± 12.8 years).

**Table 1:** Baseline characteristics of the HypoPT population (*n* = 32). Data are expressed as mean ± SD or as *n* (%).

**Characteristics**	**Values**
Gender M/F (*n*)	6/26
Age (years)	50 ± 12.7
BMI (kg/m^2^)	28.5 ± 6.9
Disease duration (years)	11.7 ± 10.5
Calcium supplementation (mg/day)	1,482 ± 955
Calcitriol (mcg/day)	0.76 ± 0.49
Cholecalciferol supplementation (IU/day)	1959.37 ± 191.52
ITEL-MMSE score	20.9 ± 1.0
ITEL-MMSE recall subscore	2.4 ± 0.7
PTH (pg/mL)	9.9 ± 6.8
TSH (mU/L)	1.5 ± 1.4
Serum calcium (mg/dL)	8.5 ± 0.6
Serum phosphate (mg/dL)	4.5 ± 0.9
25-OH vitamin D (ng/mL)	30.1 ± 9.2
24 h urinary calcium (mg/24 h)	328.6 ± 197.1
Creatinine (mg/dL)	0.8 ± 0.2
Nephrolithiasis *n* (%)	8 (25.0%)
Nephrocalcinosis *n* (%)	0 (0%)

HypoPT, hypoparathyroidism; ITEL-MMSE, Italian telephone version of the Mini-Mental State Examination; BMI, body mass index; PTH, parathyroid hormone; TSH, thyroid-stimulating hormone; and IU, international unit.

All HypoPT subjects were receiving levothyroxine therapy, and none of the patients had hyperthyroidism. At the time of baseline evaluation, all HypoPT patients were treated with calcitriol (0.76 ± 0.48 μg/die) and calcium carbonate (1,482 ± 0.955 mg). All HypoPT subjects were receiving cholecalciferol supplementation. Compared with the control group, HypoPT patients had significantly lower Alb-Ca (8.5 ± 0.6 vs 9.51 ± 0.36 mg/dL, *P* < 0.001), higher phosphate (4.5 ± 0.9 vs 3.51 ± 0.75 mg/dL, *P* < 0.001), and lower PTH (9.9 ± 6.8 vs 43.7 ± 23.3 pg/dL, *P* < 0.001). The indications for thyroidectomy in HypoPT included differentiated thyroid cancer (*n* = 16, 50%) and nontoxic goiter (*n* = 16, 50%). No significant differences in the serum thyroid function or eGFR were recorded between the two groups (Table 2).

**Table 2:** Baseline biochemical characteristics of the study population. Data are expressed as mean ± SD.

	HypoPT	Controls	*P* value
Gender M/F (*n*)	6/26	4/12	
Age (years)	50 ± 12.7	45.4 ± 12.8	0.108
PTH (pg/mL)	9.9 ± 6.8	43.7 ± 23.3	<0.001
TSH (mU/L)	1.5 ± 1.4	1.5 ± 0.53	0.461
Serum calcium (mg/dL)	8.5 ± 0.6	9.51 ± 0.36	<0.001
Serum phosphate (mg/dL)	4.5 ± 0.9	3.51 ± 0.75	<0.001
25-OH vitamin D (ng/mL)	30.1 ± 9.2	29.8 ± 9.1	0.462
Creatinine (mg/dL)	0.8 ± 0.2	0.78 ± 0.15	0.269

HypoPT, hypoparathyroidism; PTH, parathyroid hormone; and TSH, thyroid-stimulating hormone.

At 48 months following initiation of therapy in the PTH-treated subgroup, patients receiving palopegteriparatide (*n* = 3) were treated with a mean daily dose of 27 μg, while those receiving teriparatide (*n* = 3) received a mean daily dose of 40 μg. All changes in biochemical parameters before and after 48 months of PTH therapy in HypoPT patients are reported in Table 3. At the 48-month evaluation, all patients exhibited normal thyroid-stimulating hormone (TSH) levels, with a mean serum albumin-corrected calcium of 8.96 ± 0.2 mg/dL and phosphate level of 4.0 ± 0.4 mg/dL despite the complete discontinuation of calcium and active vitamin D supplements.

**Table 3:** Changes in biochemical parameters following 48 months of PTH therapy in six HypoPT patients. Data are expressed as mean ± SD.

	HypoPT before PTH therapy	HypoPT after PTH therapy	*P* value
Gender M/F (*n*)	1/5	1/5	NA
TSH (mU/L)	2.01 ± 1.4	2.54 ± 1.4	0.265
Serum calcium (mg/dL)	8.71 ± 0.23	8.96 ± 0.29	0.066
Serum phosphate (mg/dL)	4.1 ± 0.65	4.0 ± 0.4	0.362
25-OH vitamin D (ng/mL)	35.3 ± 10.7	34.6 ± 9.7	0.456
Creatinine (mg/dL)	0.82 ± 0.18	0.86 ± 0.17	0.359

HypoPT, hypoparathyroidism; PTH, parathyroid hormone; and TSH, thyroid-stimulating hormone.

### Cognitive and neurophysiological evaluation

Each participant underwent cognitive screening using the ITEL‐MMSE, with each session lasting approximately 5 min. The total mean (±SD) score on the ITEL‐MMSE was 20.96 ± 0.19 out of 22, while the mean score for the ‘recall’ item was 2.4 ± 0.7, indicating a global normal cognitive status in all patients.

Before iTBS, the mean (±SD) peak-to-peak MEP amplitude was 0.9 ± 0.7 mV for HypoPT patients and 0.8 ± 0.5 mV for healthy controls. After iTBS, the MEP amplitude was 0.9 ± 0.6 mV in HypoPT patients and 1.2 ± 0.6 mV in controls. The MEP_diff was 0.41 ± 0.4 mV for controls and −0.02 ± 0.06 mV for HypoPT patients; this difference was statistically significant (*W* = 527, *P* = 0.003) with a moderate-to-large effect size (rank-biserial correlation rrb = 0.53) (Table 4). To account for multiple comparisons, Bonferroni correction was applied. Among the tested variables, only the change in MEP amplitude before and after stimulation remained statistically significant after correction (corrected *P* = 0.042).

**Table 4:** Neurophysiological parameters of interest: comparison between healthy controls and patients with hypoparathyroidism. Data are presented as mean ± SD. Data were analyzed using the Wilcoxon test, with statistical significance set at *P* < 0.05.

	Healthy controls (*n* = 16)	HypoPT patients (*n* = 32)	*W*	*P*
MEP_pre, mV	0.8 ± 0.5	0.9 ± 0.7	364.5	0.55
MEP_post, mV	1.2 ± 0.6	0.9 ± 0.6	474	0.07
MEP_diff, mV	0.4 ± 0.4	−0.02 ± 0.06	527	0.003*
SICI	0.4 ± 0.3	0.5 ± 0.3	270.5	0.16
SAI	0.5 ± 0.2	0.6 ± 0.3	303	0.92
ICF	1.5 ± 0.5	1.4 ± 0.5	335	0.88
MEP amplitude at SICI/ICF intervals				
Baseline	1.3 ± 0.7	1.1 ± 0.6	235.5	0.54
SICI 2 ms, mV	0.4 ± 0.3	0.5 ± 0.3	193	0.51
SICI 3 ms, mV	0.6 ± 0.3	0.5 ± 0.3	262.5	0.16
ICF 15 ms, mV	1.7 ± 1.0	1.4 ± 0.6	230	0.54
MEP amplitude at SAI intervals				
Baseline	0.9 ± 0.4	0.9 ± 0.3	183	0.85
SAI 2, ms, mV	0.6 ± 0.4	0.5 ± 0.3	113.5	0.41
SAI 3, ms, mV	0.4 ± 0.2	0.5 ± 0.3	177.5	0.71
SAI 4, ms, mV	0.5 ± 0.3	0.6 ± 0.4	188.5	0.98

* This finding remained statistically significant after Bonferroni correction (corrected *P* = 0.042).

HypoPT, hypoparathyroidism; MEPs, motor evoked potentials; SICI, short-interval intracortical inhibition; SAI, short-latency afferent inhibition; and ICF, intracortical facilitation.

Regarding the other TMS parameters, we calculated the ratio of conditioned MEP (for all ISIs in each protocol: SICI, ICF, and SAI) to unconditioned MEP. The SAI ratio was 0.6 ± 0.3 for HypoPT patients and 0.5 ± 0.3 for controls, the SICI ratio was 0.5 ± 0.3 in HypoPT patients and 0.4 ± 0.3 in controls, and the ICF ratio was 1.4 ± 0.5 for HypoPT patients and 1.5 ± 0.5 for controls. Mean MEP amplitude values for each interval and condition are reported in Table 4. No significant differences in SAI, SICI, or ICF were observed between HypoPT patients and healthy controls (Table 4). No significant correlations between neurophysiological and biochemical parameters were observed.

In the subgroup of six HypoPT patients treated with PTH therapy, we repeated the neurophysiological evaluation 48 months after the initiation of treatment. TMS-related parameters and the comparisons before and after the initiation of PTH therapy are reported in Table 5.

**Table 5:** Changes in neurophysiological parameters following PTH therapy in six HypoPT patients. Data are presented as mean ± SD. Data were analyzed using the Wilcoxon test, with statistical significance set at *P* < 0.05. Statistically significant values are presented in bold.

	Before PTH therapy	After PTH therapy	*W*	*P*
MEP_pre, mV	1.46 ± 0.6	1.12 ± 0.7	15	0.699
MEP_post, mV	1.11 ± 0.7	1.43 ± 0.6	20	0.818
MEP_diff	−0.35 ± 0.5	0.30 ± 0.3	31	**0.041**
SICI	0.44 ± 0.2	0.38 ± 0.2	14	0.589
SAI	0.46 ± 0.3	0.36 ± 0.1	18	1.000
ICF	1.57 ± 0.5	1.63 ± 0.5	20	0.818
MEP amplitude at SICI/ICF intervals				
Baseline, mV	1.08 ± 0.5	1.36 ± 0.5	23	0.485
SICI 2 ms, mV	0.44 ± 0.2	0.57 ± 0.6	17	0.937
SICI 3 ms, mV	0.39 ± 0.2	0.47 ± 0.5	19	0.937
ICF 15 ms, mV	0.42 ± 0.1	0.50 ± 0.3	21	0.699
MEP amplitude at SAI intervals				
Baseline, mV	1.12 ± 0.5	0.99 ± 0.3	17	0.937
SAI 2 ms, mV	0.47 ± 0.3	0.31 ± 0.2	10	0.410
SAI 3 ms, mV	0.44 ± 0.2	0.36 ± 0.2	14	0.589
SAI 4 ms, mV	0.44 ± 0.3	0.48 ± 0.3	16	0.818
Average SAI, mV	0.47 ± 0.2	0.37 ± 0.1	13	0.485

HypoPT, hypoparathyroidism; MEPs, motor evoked potentials; SICI, short-interval intracortical inhibition; SAI, short-latency afferent inhibition; and ICF, intracortical facilitation.

In particular, we observed a significant increase in MEP_diff (−0.35 ± 0.5 mV before replacement therapy; 0.3 ± 0.3 mV after the replacement therapy; *W* = 31, *P* = 0.04), while the remaining parameters remained unchanged.

## Discussion

In the present study, for the first time, we have assessed cortical excitability and plasticity in a cohort of patients with HypoPT using various TMS protocols. Our findings indicate that HypoPT patients exhibit a selective impairment in plasticity propensity compared to age- and sex-matched healthy controls and that PTH therapy may reverse this functional alteration.

It is well known that HypoPT is associated with measurable changes in brain structure (12) and cognitive performances (13); however, the underlying mechanisms are still not clear. A direct effect of PTH has been proposed since the expression of the PTH receptors in the brain is well documented (29, 30). Notably, PTH receptors are particularly expressed in brain areas related to memory and learning, such as hippocampus and cerebral cortex (30), and HypoPT patients with cognitive deficit exhibit structural and functional alterations in these areas on neuroimaging studies. Indeed, Sikjaer *et al.* (12) found a reduction in hippocampal and thalamic volumes using MRI, while Terada *et al.* showed a significant reduction in 18-FDG uptake in bilateral frontal, left temporal, and parietal cortices, using PET (31).

In this study, we employed neurophysiology to explore cortical excitability and plasticity propensity in HypoPT patients (25). We found that the response to iTBS differed significantly between patients and controls and that PTH therapy could normalize this response.

iTBS is a rTMS protocol in which magnetic pulses are repeatedly delivered to a targeted brain region, inducing lasting excitatory effects on cortical excitability, consisting in a sustained and prolonged increase in MEP amplitude after stimulation (28). It is widely demonstrated that this effect depends on synaptic plasticity phenomena, namely LTP (32). Briefly, LTP occurs through the activation of glutamatergic N-methyl-D-aspartate (NMDA) receptors at the postsynaptic terminal, allowing calcium (Ca^2+^) influx into the neuron. The increased intracellular Ca^2+^ concentration triggers several signaling pathways, including the activation of calcium/calmodulin-dependent protein kinase II (CaMKII). This, in turn, enhances the number and sensitivity of membrane receptors, further facilitating calcium influx. Over time, gene expression and protein synthesis lead to structural modifications of synapses, such as dendritic spine enlargement and increased receptor density, resulting in a persistent increase in synaptic strength (33, 34).

In our study, we found that, as expected (35), healthy subjects have an increase in MEP amplitude after iTBS. Instead, in HypoPT patients, iTBS failed to produce changes in MEP amplitude, thus suggesting that HypoPT is associated with functional alteration of LTP mechanisms. This idea is further supported by the effect of PTH therapy that was associated with normalization of plasticity measures in this exploratory subgroup. The response to TBS is well established in the literature as one of the most extensively studied TMS protocols (27). Therefore, the lack of potentiation observed in our study represents an interesting finding, even in the absence of changes in other, unrelated TMS parameters.

Despite pharmacokinetic differences, both palopegteriparatide and teriparatide are capable of activating PTH receptors, including PTH2R, which is expressed in specific brain regions and may play a role in neurophysiological regulation. Their combined analysis reflects a shared theoretical mechanism of action relevant to the study aims. Indeed, in patients treated with PTH therapy, the iTBS-induced changes in MEP amplitude are similar to those observed in healthy controls.

PTH may act on synaptic mechanisms directly (36) or, more likely, indirectly by modulating calcium dynamics. Although our study population exhibited normal serum calcium levels, we cannot exclude the possibility that calcium homeostasis – particularly in the synaptic cleft – may be altered in cases of HypoPT, especially in processes occurring on a millisecond timescale, such as synaptic functionality. Even slight alterations of calcium signaling could result in subtle dysfunction of the synaptic machinery, potentially leading to less effective plasticity phenomena. The essential role of calcium in synaptic plasticity has been well established for decades (37). Some evidence suggests that calcium concentration dynamics – in terms of magnitude and duration of calcium influx in the post-synaptic terminal – might have a direct influence on LTP induction. According to the calcium-control hypothesis, the level and duration of postsynaptic calcium transients actually determine whether a synapse undergoes long-term plastic changes (38). In this context, even subtle alterations in the spatial and temporal regulation of synaptic calcium microdomains could interfere with NMDA receptor-dependent LTP induction. Since iTBS probes LTP-like plasticity through NMDA-dependent calcium-mediated mechanisms, it may be particularly sensitive to such dysfunction, potentially explaining the blunted plastic response observed in our HypoPT cohort.

Synaptic plasticity dysfunction is increasingly recognized as a critical factor in the pathogenesis of cognitive impairment (39). In Alzheimer’s disease, the most common neurodegenerative cause of cognitive dysfunction, the LTP-like plasticity impairment, assessed through TMS, is considered an early neurophysiological disease hallmark that can be already present in the prodromal phase and correlates with disease progression (20), while in more advanced stages, also SAI and motor thresholds are altered, indicating a more diffuse damage (20).

Interestingly, in our study, we found that response to iTBS is altered in HypoPT patients with normal ITEL-MMSE scores, thus suggesting that such a functional impairment could be a neurophysiological feature of all HypoPT patients, even those without a clinically evident cognitive impairment. Cognitive evaluation in our study was limited to the ITEL-MMSE, a screening tool primarily designed to detect global cognitive impairment. Therefore, subtle executive dysfunction, attentional deficits, or alterations in processing speed cannot be excluded and should be investigated in future studies using comprehensive neuropsychological batteries.

This hypothesis is in line with previous studies showing that PTH influences synaptic activity also in physiological conditions (40); thus, it is likely that an abnormal interaction between PTH and its receptors as observed in HypoPT patients could produce a change in LTP mechanisms, even in the absence of clinical manifestations.

In other terms, similarly to Alzheimer’s disease where neuronal hyperexcitability (41) and synaptic dysfunction (42) may be detectable even in the preclinical stage of the disease, also in HypoPT, LTP-like plasticity impairment could represent an early pathogenetic mechanism of CNS involvement, before overt clinical symptoms emerge. Further studies are needed to confirm this hypothesis and to identify disease and/or individual factors that can facilitate progression to clinically evident cognitive impairment in some patients. To this purpose, it is mandatory, in future studies, to evaluate TMS measures of cortical plasticity and excitability also in HypoPT patients with cognitive impairment and to use more comprehensive and detailed neuropsychological tests for cognitive evaluation.

In the present study, we enrolled patients without overt neuropsychiatric disorders and with normal ITEL-MMSE scores; however, we acknowledge that a comprehensive neuropsychological assessment was not performed and may have revealed subtle cognitive dysfunction.

Other limitations of our study are as follows: i) the calcium level was measured only once during the study; ii) we did not perform neuroimaging studies to identify brain calcifications or other brain structural changes that can influence cortical plasticity and excitability; iii) a longitudinal follow-up of conventionally treated patients was lacking; iv) PTH-treated subjects were given both teriparatide and palopegteriparatide that differ in pharmacokinetics, approved dosing, and clinical use, and without direct data currently supporting equivalence of their central neurophysiological effects, and v) although we relied exclusively on well-established TMS protocols, such as iTBS – whose role in assessing plasticity is extensively supported by the existing literature – we acknowledge that these measures are not yet validated for routine clinical practice. Therefore, on this point, our findings should be considered exploratory and hypothesis-generating.

On the other hand, this study has several strengths: i) biochemical and neurophysiological assessments were performed in a single center; ii) for the first time, cortical excitability, circuit functioning, and plasticity propensity were investigated in HypoPT patients in comparison with controls; and iii) in a subgroup of patients, we evaluated the effects of PTH therapy on objective, neurophysiological parameters.

Our work has important potential clinical implications. Conventional neuropsychological tests are sometimes inadequate to catch the cognitive impairment – e.g. ‘brain fog’ and ‘slow thinking” – HypoPT patients complain about. Thus, a gap remains between the subjective symptoms and our ability to detect them. Neurophysiological techniques – especially TMS, giving its potential for evaluating non-invasively plasticity propensity – can fill this gap. In this context, early recognition and monitoring of synaptic dysfunction in HypoPT patients might serve as a biomarker to monitor and guide PTH replacement therapy. Moreover, since TMS is less invasive and less expensive than other techniques, such fMRI, it is ideal for exploring the pathogenetic mechanisms of cognitive impairment and for testing the effect of pharmacological interventions. In addition, TMS represents a powerful means to probe *in vivo* the synaptic function and plasticity at different disease stages even in the very early phases when there are no structural changes, and at the same time, it may be used as a therapeutic tool to modulate maladaptive plasticity in patients. Functional data on neuronal networks activated by TMS can be usefully integrated with information obtained by more standardized diagnostic approaches, such as amyloid-PET or CSF-amyloid tau levels. Moreover, TMS, being less invasive and less expensive than other techniques, is ideal for testing in ‘real time’ pharmacological and non-pharmacological interventions at synaptic level and for serial evaluation of patients with cognitive impairment.

In conclusion, our findings suggest that HypoPT patients present a functional impairment of neural plasticity mechanisms, in the absence of overt cognitive impairment on screening assessment. PTH therapy seems to restore this impairment, but we believe that further comprehensive and prospective studies with long-term follow-up are required to fully show the clinical reflection of our research and the correlation between biochemical and clinical parameters.

## Declaration of interest

AP served as a consultant for Ascendis, and he received travel grant from Ascendis. AMN received travel grant from Ascendis. All other authors state that they have no conflicts of interest. AP is a Senior Editor of *Endocrine Connections*. AP was not involved in the review or editorial process for this paper, on which he is listed as an author.

## Funding

This research did not receive any specific grant from funding agencies in the public, commercial, or not-for-profit sectors.

## Author contribution statement

AP conceived the study, designed the methodology, supervised the study, and wrote, reviewed, and edited the manuscript. GT performed investigation, curated the data, and wrote, reviewed, and edited the manuscript. AMN performed investigation, curated the data, and prepared the original draft of the manuscript. ES, MC, and FM curated the data and wrote, reviewed, and edited the manuscript. MR, AT, DN, LMC, GMR, and GM performed investigation and curated the data. AC performed investigation and wrote, reviewed, and edited the manuscript. MVDiL curated the data and wrote, reviewed, and edited the manuscript. FC designed the methodology, supervised the study, and wrote, reviewed, and edited the manuscript.

## Data availability

Some or all datasets generated during and/or analyzed during the current study are not publicly available but are available from the corresponding author on reasonable request.
